# Efficiency and safety of ablation procedure for the treatment of atrial fibrillation in valve surgery: A PRISMA-compliant cumulative systematic review and meta-analysis

**DOI:** 10.1097/MD.0000000000028180

**Published:** 2021-12-17

**Authors:** Tianyao Zhang, Xiaochu Wu, Yu Zhang, Lin Zeng, Bin Liu

**Affiliations:** a Department of Anesthesiology, the First Affiliated Hospital of Chengdu Medical College, Sichuan, China; b National Clinical Research Center for Geriatrics and Department of Anesthesiology, West China Hospital, Sichuan University, Chengdu, Sichuan Province, China.

**Keywords:** ablation, atrial fibrillation, meta-analysis, RCT, valve surgery

## Abstract

**Background:**

Atrial fibrillation is the main complication of patients who suffer from valvular heart disease (VHD), which may lead to an increased susceptibility to ventricular tachycardia, atrial dysfunction, heart failure, and stroke. Therefore, seeking a safe and effective therapy is crucial in prolonging the lives of patients with VHD and improving their quality of life.

**Methods:**

Our target database included PubMed, Web of Science, Embase, and Cochrane Library, from which published articles were retrieved from inception to June 2020. We retrieved all randomized controlled trials (RCTs) that compared patients undergoing valve surgery with (VSA) or without ablation (VS) procedure. Studies to be included were screened and data extraction was performed independently by 2 investigators. The Cochrane risk-of-bias table was used to evaluate the methodological quality of the included RCTs. The mean difference (MD) with 95% confidence interval (CI) and relative risk (RR) ratio was calculated to analyze the data. Heterogeneity was evaluated using I^2^ and chi-square tests. Egger test and the trim and fill analysis were used to further determine publication bias.

**Results:**

Fourteen RCTs that included 1376 patients were eventually selected for this meta-analysis. Surgical ablation was found to be effective in restoring sinus rhythm in valvular surgery patients at discharge (RR 2.91, 95% CI [1.17, 7.20], I^2^ 97%, *P* = .02), 3 to 6 months (RR 2.85, 95% CI [2.27, 3.58], I^2^ 49%, *P* < .00001), 12 months, and more than 1 year after surgery (RR 3.54, 95% CI [2.78, 4.51], I^2^ 27%, *P* < .00001). All-cause mortality (RR 0.98, 95% CI [0.64, 1.51], I^2^ 0%, *P* = .94) and stroke (RR 1.29, 95% CI [0.70, 2.39], I^2^ 0%, *P* = .57) were similar in the VSA and VS groups. Compared with VS, VSA prolonged cardiopulmonary bypass time (MD 30.44, 95% CI [17.55, 43.33], I^2^ 88%, *P* < .00001) and aortic cross-clamping time (MD 19.57, 95% CI [11.10, 28.03], I^2^ 89%, *P* < .00001). No significant differences were found between groups with respect to the risk of bleeding (RR 0.64, 95% CI [0.37, 1.12], I^2^ 0%, *P* = .12), heart failure (RR 1.11, 95% CI [0.63, 1.93], I^2^ 0%, *P* = .72), and low cardiac output syndrome (RR 1.41, 95% CI [0.57, 3.46], I^2^ 18%, *P* = .46). However, the demand for implantation of a permanent pacemaker was significantly higher in the VSA group (RR 1.84, 95% CI [1.15, 2.95], I^2^ 0%, *P* = .01).

**Conclusion:**

Although we found high heterogeneity in the restoration of sinus rhythm at discharge, we assume that the comparison is valid at this time, given the current state in the operating room. This study provides evidence of the efficacy and security of concomitant ablation intervention for patients with VHD and atrial fibrillation. Surgical ablation would increase the safety of implantation of a permanent pacemaker in the population that underwent valve surgery.

## 1 Introduction

Atrial fibrillation (AF) is the most common arrhythmia encountered in clinical practice and is a serious and frequent problem affecting 30% to 50% of patients with valvular heart disease (VHD). A statistics report by the Global Health Data Exchange database shows that about 37,574 million individuals (0.51% of the worldwide population) suffer from AF, which is an increase of 33% compared with the past 20 years. This burden is projected to increase by more than 60% in 2050. VHD is a prevalent condition characterized by stenotic and regurgitant lesions of heart valves, which may affect a patient's quality of life. Currently, there are only limited therapeutic options to manage this condition. Heart valve surgery is often the only option to enhance long-term survival of patients with this condition. After cardiac surgery, especially valvular surgery, postoperative AF is one of the most common complications and has a reported incidence of 10% to 50%. Recently, several retrospective studies have reported that surgical ablation could improve long-term survival in patients who underwent cardiac surgery.^[1–4]^ However, a report by Mehaffey et al^[5]^ in 2020 generated from the Regional Society of Thoracic Surgeons database demonstrated that the use of concomitant AF ablation decreased from 2011 to 2018 at an annual rate of 2.82%. This finding might be a result of the ambiguity of the therapeutic effect of surgical ablation in the view of the surgeon. Previous studies have confirmed the evaluation of the efficacy of cardiac surgery with concurrent ablation,^[6]^ comparison of the efficiency of pulmonary vein isolation and maze surgery,^[7]^ comparison of different ablation energy effects,^[8]^ and comparison of left atrial and biatrial maze procedure.^[9]^ However, there is a dearth of special, high-quality systematic reviews that evaluate the efficacy and security of surgical ablation in patients with VHD who underwent valvular surgery. Given the hesitation or its lack thereof in choosing ablation, the present cumulating meta-analysis sought to assess current evidence based on reported studies.

## 2 Methods

### 2.1 Literature search

A comprehensive literature search strategy was conducted to identify randomized controlled trials (RCTs) suitable for inclusion in this systematic review. We searched published studies from the date of inception to June 2020 in PubMed/Medline, Embase, Web of Science, and Cochrane Central Register of Controlled Trials. ClinicalTrials.gov was also searched to stay abreast with the ongoing clinical trials. To achieve maximum sensitivity of the search strategy, we combined variants of the terms “atrial fibrillation” OR “AF” AND “ablation” OR “Maze Procedure” AND “valve surgery” OR “valvular surgery” OR “valve replacement” as either keywords or MeSH terms (search strategy details are listed in supplementary A). All retrieved references were reviewed based on their inclusion and exclusion criteria to assess their suitability to be included in this meta-analysis.

### 2.2 Selection criteria

The inclusion criteria to be included in this systematic review and meta-analysis were as follows:

1. RCTs
2. Patients concomitant with persistent or long-standing persistent AF who underwent heart valve surgery
3. Patients between 18 and 80 years of age
4. Surgery ablation energy was unlimited
5. Studies that made a direct comparison between valve surgery with or without surgical ablation
6. Study endpoints were sinus rhythm (SR) or AF-free survival.

The exclusion criteria for this systematic review and meta-analysis were as follows:

1. Studies that were not RCTs, for example, retrospective study, cohort study
2. Studies that used maze ablation without concomitant valve surgery, or those that used only valve surgery without maze ablation
3. Studies comparing other ablation techniques or different ablation energy
4. Studies involving surgery to other parts of the heart
5. Duplicate data from the same study
6. Studies not including a control group comprising patients who only underwent valve surgery
7. Studies from which data could not be extracted or merged

### 2.3 Data extraction

All retrieved articles were reviewed by 2 investigators (TZ and XW). All data from the texts, tables, and figures were extracted and compiled by 2 investigators (TZ and XW). The extracted information included study type, sample size, participant demographics and baseline characteristics, details of the intervention and control conditions, outcomes and times of measurement, and information on the assessment of the risk of bias. Discrepancies were resolved by discussion and consensus with a third investigator (BL). The Cochrane risk bias assessment table was used to assess the risk bias of the included studies and was completed by LZ and YZ. Funnel plots and Egger test were used to assess publication bias for the efficacy and safety of the ablation procedure for the treatment of AF in valve surgery. The final results were reviewed by the senior investigators (BL and LZ).

### 2.4 Data synthesis

A structured data collection report was prepared to derive the following information from each study: title, name of the first author, year of study and publication name, country where the study was conducted, subject demographics, ablation procedure used, time for cardiopulmonary bypass (CPB) and aortic valve block, and electrocardiography monitoring. Primary outcomes included maintenance of SR and recurrence of AF. Secondary outcomes included stroke, death, early and late heart failure, low cardiac output syndrome, bleeding, and pacemaker implantation. This study was conducted according to the Preferred Reporting Items for Systematic Reviews and Meta-Analysis (PRISMA) guidelines.^[11]^

### 2.5 Ethical approval and patient consent

This is a meta-analysis based on RCTs which have been published, so the ethical approval and patient consent is not applicable for our study.

### 2.6 Statistical analyses

Revman 5.3 was used to process the data from included RCTs. The mean difference (MD) with 95% confidence interval (CI) and relative risk (RR) ratio was calculated to analyze the enumeration data. I^2^ and chi-square tests were used to determine whether the results of the included studies were heterogeneous. The heterogeneity was small if *P* > .05 and I^2^ ≤ 50%, and the fixed-effect model was used for analysis. If *P* ≤ .05 and I^2^ > 50%, the statistical heterogeneity between the 2 groups was considered and the causes of heterogeneity were analyzed. Descriptive analysis was carried out if there was obvious clinical heterogeneity and the random-effect model was used for analysis. Publication bias of the major outcomes of this meta-analysis was determined using Egger test. Trim and fill analysis was performed to adjust for publication bias. All *P* values were two-sided and all statistical analyses were conducted using Review Manager Version 5.3 (Cochrane Collaboration, Software Update) and R.

## 3 Results

### 3.1 Literature search

**Figure 1:**
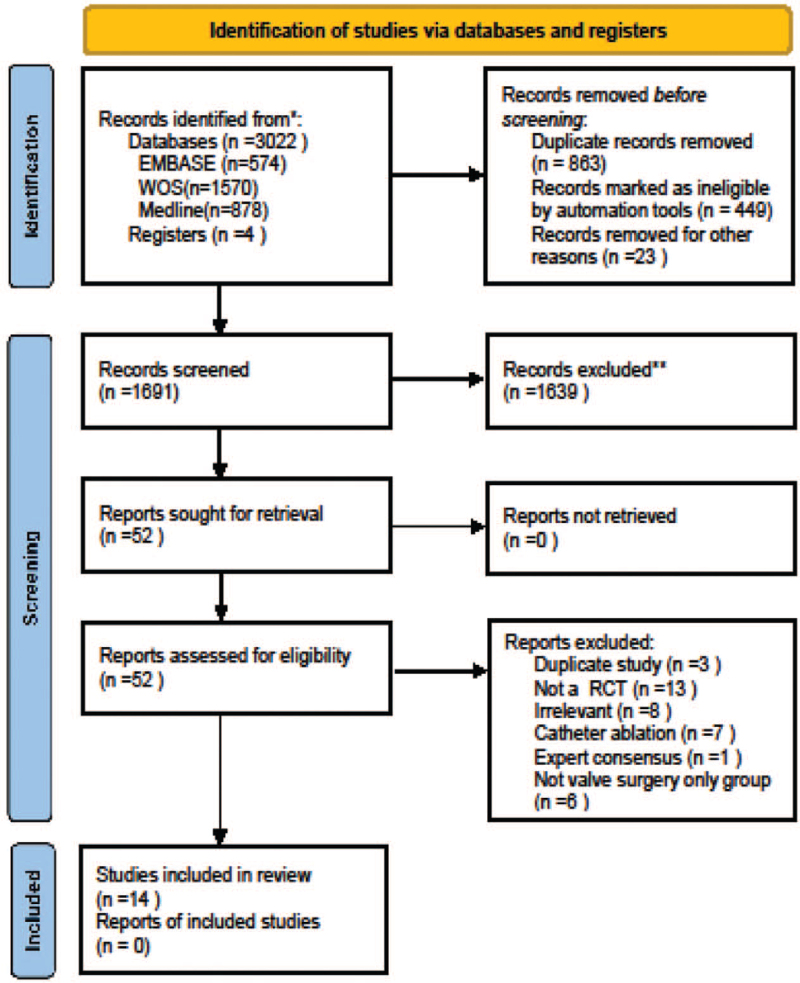
Flow diagram of literature retrieval.

A total of 3185 studies were retrieved from our target electronic databases. After the exclusion of duplicate or irrelevant references, 1691 potentially relevant articles were retrieved. After a detailed evaluation of these articles, 52 studies were selected for further assessment. After applying the selection criteria, 14 RCTs were selected for analysis (see Fig. 1). In these 14 studies, 1376 patients underwent procedures that involved valve surgery with surgical ablation (VSA group; n = 712) or valve surgery without surgical ablation (VS group; n = 548). The characteristics of these RCTs are summarized in Table 1.

**Table 1:** Characteristics of included RCTs.

First author	Year	Institution	VS	VS+SA	Surgery type	Ablation type	Monitoring
Wang^[10]^	2018	Shenyang Northern Hospital (Liaoning, China)	65	65	mvr, mvr+tv	CS	ECG, 24h holter
Bagge^[11]^	2018	Uppsala University (Uppsala, Sweden)	30	35	mv	Cryoablation	ECG
Gillinov^[12]^	2015	Cleveland Clinic Foundation (Cleveland, USA)	127	133	mv,tv,av,cabg	Cryoablation	72h holter
Wang^[13]^	2014	Fuwai Hospital (Beijing, China)	70	140	mvr,avr,tv,cabg	RF	echo, ECG, holter
Von Oppell^[14]^	2009	University Hospital Wales (Cardiff, UK)	25	24	mv,tv,av,cabg	RF	ECG, echo
Chevalier^[15]^	2009	Hôpital Louis Pradel (Louis-Pradel, France)	22	21	mv,av,tv	RF	Holter
Albrecht^[16]^	2009	Fundação Universitária de Cardiologia (Porto Alegre, Brazil)	20	40	mv,av,tv	PVI, CS	ECG, echo, treadmill stress test, Doppler unidimensional
Srivastava^[17]^	2008	King Edward Memorial Hospital (Mumbai, India)	40	80	mv,tv	RF, cryoablation	ECG, 2D echo
Doukas^[18]^	2005	Glenfield Hospital (Leicester, England)	48	49	mv,tv	RF	ECG, 24h holter, Shuttle-Walk test
Abreu Filho^[19]^	2005	University of São Paulo Medical School (São Paulo, Brazil)	28	42	mv,tv	RF	24h-ECG
de Lima^[20]^	2004	Fundação Universitária de Cardiologia (Porto Alegre, Brazil)	10	20	mv,av,tv	CS	24h-holter-ECG, echo, exercise testing
Akpinar^[21]^	2003	Florence Nightingale Hospital (Istanbul, Turkey)	34	33	mv	RF	Pacemaker, 24h holter
de Vasconcelos^[22]^	2004	Instituto do Coracao (São Paulo, Brazil)	14	15	MV,TV	CS	ECG, echo
Deneke^[23]^	2002	Bergmannsheil University Hospital (Bochum, Germany)	15	15	mv	RF	Holter-ECG, echo

ECG = electrocardiography, RCTs = randomized controlled trials, VS = valvular surgery.

### 3.2 Quality of study

All of the included studies were RCTs (level 1 evidence).^[10–23]^ Nine studies had enrolled more than 50 patients (range, 60–260 patients),^[10–13,16–19,21]^ whereas only 5 studies had fewer than 50 patients (range, 29–49 patients).^[14,15,20,22,23]^ Eight studies used radiofrequency ablation,^[13–15,17–19,21,23]^ 3 studies used cryoablation,^[11,12,17]^ 4 studies used Cox-Maze cut-and-sew,^[10,16,20,22]^ and only 1 study reported patients undergoing pulmonary vein isolation.^[16]^ Permanent AF; persistent AF; and a mixture of permanent, persistent, and paroxysmal AF populations were evaluated in 7,^[11,14–16,19,20,23]^ 5,^[10,12,14,21,22]^ and 1 study,^[18]^ respectively. One of the studies reported a follow-up of greater than 3 years (60 months).^[16]^ Two studies reported follow-up between 2 and 3 years (range, 26–29 months),^[22,23]^ whereas 11 studies reported a follow-up of less than 2 years (6–18 months).^[10–15,17–21]^

**Figure 2:**
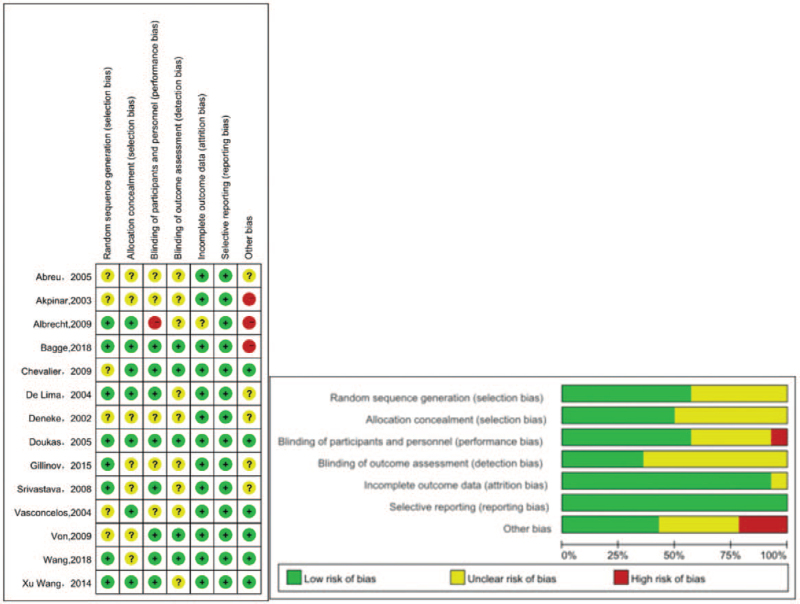
(A) Risk of bias graph: reviews of authors’ judgments about each risk of bias item are presented as percentages across all included studies. (B) Risk of bias summary: review of authors’ judgments about each risk of bias item for each included study.

SR was the primary endpoint in 8 studies,^[11,14–16,18–20,23]^ whereas AF-free survival was the primary endpoint in 6.^[10,12,13,17,21,22]^ Outcomes of SR at discharge, 3 to 6 months, and ≥1 year were reported in 9,^[14–18,20–23]^ 10,^[13–15,17–23]^ and 11 studies,^[13–23]^ respectively. AF recurrence at discharge, 3 to 6 months, and ≥1 year were reported in 5,^[13,16–19]^ 3,^[13,17,19]^ and 7 studies,^[12–14,16,17,19,20]^ respectively. The quality of the 14 RCTs was assessed using the Cochrane Collaboration by determining the risk of bias. A graph and summary of selection bias, performance bias, detection bias, attrition bias, reporting bias, and other biases identified for each RCT are shown in Figure 2.

### 3.3 Baseline and perioperative characteristics

As a companion to the progress of valvular diseases, cardiac load had some extent of variation, especially volume load, leading to an eventual deterioration in cardiac function, of which left ventricular ejection fraction (LVEF) was a sensitive signature. Pre-operative LVEF was also a key factor in postoperative outcomes. According to the pre-operative information of 805 participants from 11 studies, we found no significant differences in LVEF between the 2 groups, which indicated similar basic heart function (MD –0.52, 95% CI [–1.55, 0.52], I^2^ 0%, *P* = .33). No significant heterogeneity nor publication bias was detected (Table 2).

**Table 2:** Summary of clinical outcomes using standard meta-analysis techniques.

	n			Heterogeneity		Test for overall effect	
Outcome	VS+ ablation	VS	OR (95% CI)	*P*	I^2^	Z	*P*
SR at discharge	332	223	8.02 [3.38, 19.03]	.006	0.65	4.72	<.00001
SR at 3–6 mos	472	298	6.11 [4.31, 8.66]	.05	0.47	10.16	<.00001
SR ≥12 mos	510	316	9.92 [6.76, 14.56]	.22	0.23	11.72	<.00001
AF at discharge	316	202	0.41 [0.33, 0.51]	.02	0.7	7.8	<.00001
AF at 3–6 mos	300	180	0.43 [0.35, 0.54]	.27	0.24	7.54	<.00001
AF ≥12 mos	489	293	0.38 [0.32, 0.44]	.1	0.44	11.35	<.00001
CPB time	535	426	30.44 [17.55, 43.33]	<.00001	0.88	4.63	<.00001
AOC time	546	438	19.57 [11.10, 28.03]	<.00001	0.89	4.53	<.00001
Pre-operative LVEF	444	361	–0.52 [–1.55, 0.52]	.84	0	0.97	.33
Permanent pacemaker	712	532	1.97 [1.18, 3.29]	.66	0	2.59	.01
Bleeding	496	382	0.62 [0.34, 1.14]	.79	0	1.53	.13
Stroke	476	392	1.24 [0.64, 2.41]	.66	0	0.64	.52
Death	752	552	0.87 [0.57, 1.31]	.69	0	0.68	.5
Heart failure	281	275	1.12 [0.60, 2.07]	.59	0	0.35	.72
Low cardiac output syndrome	268	188	1.43 [0.55, 3.74]	.29	0.2	0.73	.47

AF = atrial fibrillation, AOC = aortic cross-clamp, CI = confidence interval, CPB = cardiopulmonary bypass, LVEF = left ventricular ejection fraction, OR = odds ratio, SR = sinus rhythm.

CPB was the crucial step in the entire surgery. Due to the additional procedure, the time for CPB and aortic cross-clamp (AOC) was extremely prolonged in the VSA group (CPB: MD 30.44, 95% CI [17.55, 43.33], I^2^ 88%, *P* < .00001; AOC: MD 19.57, 95% CI [11.10, 28.03], I^2^ 89%, *P* < .00001), and high heterogeneity was found. Therefore, a sensitivity analysis was performed, which demonstrated that omitting any one of included studies did not lead to a change in *P* value and maintained an I^2^ value higher than 80%. The high risk of heterogeneity did not impact the final results. Egger test score (*P* = .1611) revealed the absence of obvious publication bias.

Six studies demonstrated that the total length of hospitalization was not significantly different between the VSA and VS groups (MD –0.09, 95% CI [–1.05, 0.87], I^2^ 0%, *P* = .86). No publication bias and heterogeneity were observed.

### 3.4 Efficiency in restoring SR

**Figure 3:**
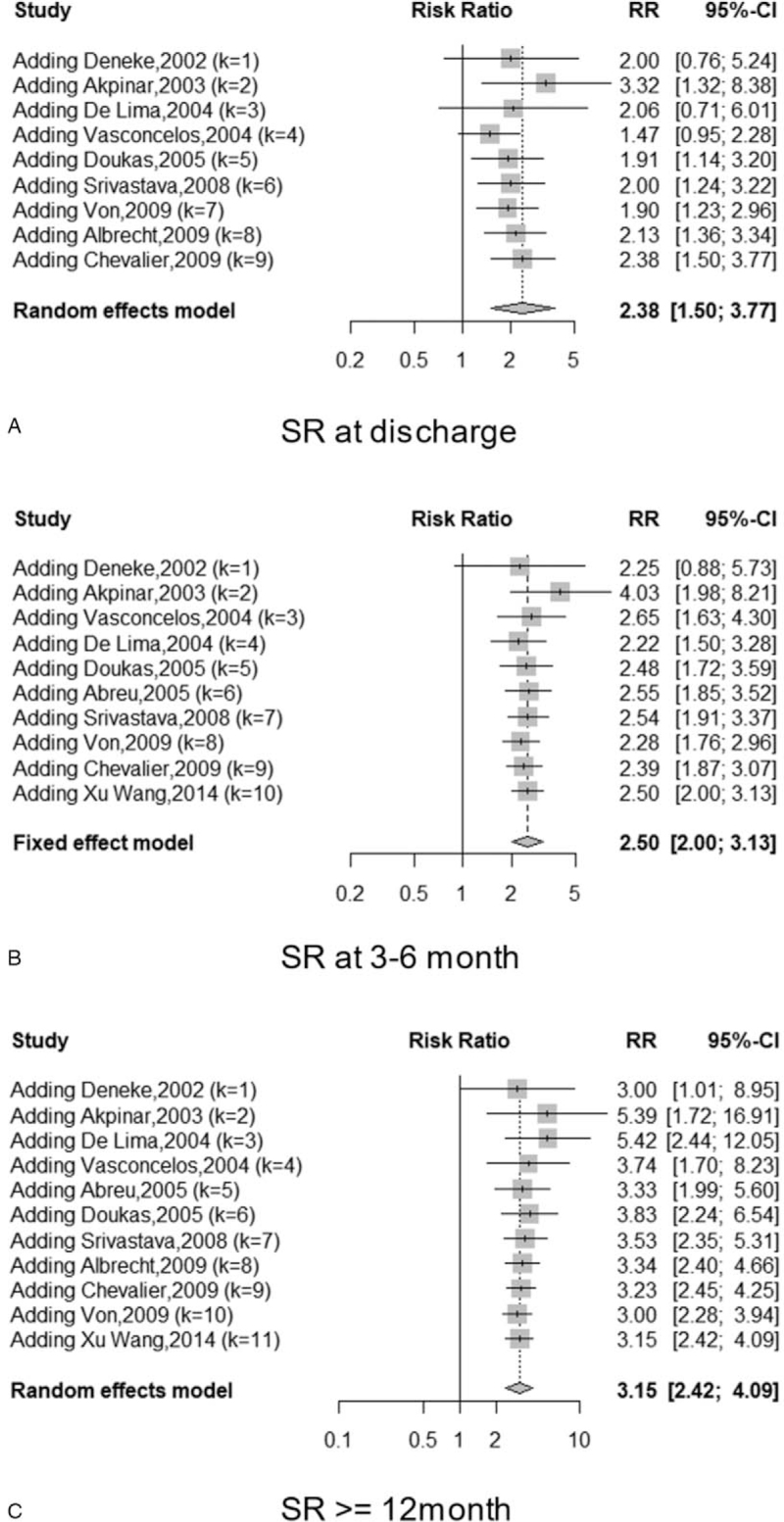
Cumulative forest plots of the RR of restoration of SR (A) at discharge, (B) 3 to 6 months, (C) 12 months and more than 12 months follow-up. RR = relative risk, SR = sinus rhythm.

We identified 11 RCTs including 826 patients that elucidated the restoration of beat rhythm postoperatively at 12 months and beyond 1 year. The maintenance of SR in the VSA group was higher than that in the VS group (62.3% vs 18%, RR 3.54, 95% CI [2.78, 4.51], I^2^ 27%, *P* < .00001). In the VSA group, 318 out of 510 individuals maintained SR, whereas only 57 out of 316 maintained SR in the VS group. Going back further from the earlier time of operation, we found that 62.7% and 22.9% of patients achieved SR at discharge in the VSA group and VS group, respectively (RR 2.91, 95% CI [1.17, 7.20], I^2^ 97%, *P* = .02). At 3 to 6 months after surgery, the VSA group had a higher number of individuals in whom the SR was restored compared with those in the VS group (61% vs 21.5%, RR 2.85, 95% CI [2.27, 3.58], I^2^ 49%, *P* < .00001). The cumulative meta-analysis demonstrated that earlier studies had higher heterogeneity and preferred surgical ablation over valve surgery only with respect to SR restoration (see Fig. 3). Egger test to determine publication bias in restoring SR confirmed significant bias at discharge (*P* < .001), but insignificant bias at 3 to 6 months (*P* = .135), and at 12 months and more than 1 year (*P* = .056). As for SR at discharge, the heterogeneity of studies was higher than 50% at discharge; therefore, we conducted a sensitivity analysis. When omitting each study one at a time, the I^2^ did not change considerably and the *P* value was significant. Therefore, this heterogeneity had no influence on the final results. The observed heterogeneity might have resulted from the several tools used to measure SR, especially in the study by Akpinar et al,^[21]^ which used data to assess heart rhythm based on pacemaker use, thereby contributing the most heterogeneity to this outcome.

### 3.5 Assessment of safety

#### 3.5.1 All-cause mortality

**Figure 4:**
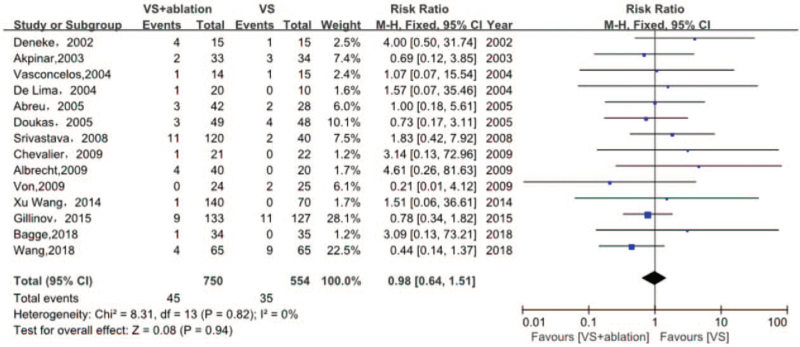
Forest plot of all-cause death after surgery, showing summary of RR with 95% CI for included studies. CI = confidence interval, RR = relative risk, VS = valvular surgery.

Fourteen studies including 1304 participants contributing to all-cause mortality showed that there were no significant differences between the 2 groups (RR 0.98, 95% CI [0.64, 1.51], I^2^ 0%, *P* = .94) (Fig. 4). Funnel plots did not reveal significant asymmetry, and the linear regression analysis conducted using Egger test showed no obvious publication bias (t = 1.7189, df = 12, *P* = .1113). Four missing studies were suggested using the trim and fill method, yet the overall effect was unchanged when these 4 studies were added (RR 0.7214, 95% CI [0.4921, 1.0576], I^2^ 0%, *P* = .0943). Sensitivity analysis did not reveal any particular study contributing to heterogeneity. In other words, significant publication bias did not exist in all-cause mortality.

### 3.6 Morbidity of stroke

**Figure 5:**
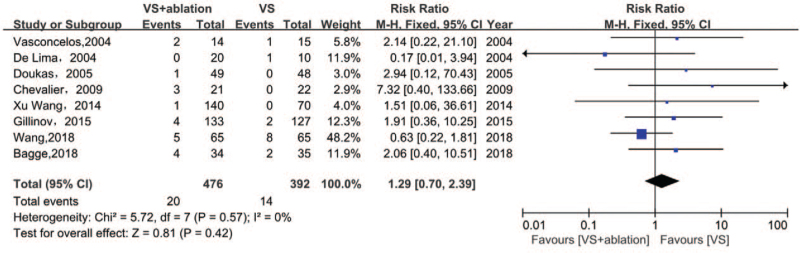
Forest plot of the incidence of stroke after surgery, showing summary of RR with 95% CI for included studies. CI = confidence interval, RR = relative risk, VS = valvular surgery.

Morbidity of stroke was reported by 8 studies that enrolled 868 patients (Fig. 5). There were no significant differences in postoperative stroke irrespective of surgical ablation (RR 1.29, 95% CI [0.70, 2.39], I^2^ 0%, *P* = .57) (Fig. 5). No obvious heterogeneity was observed in this comparison. Although the trim and fill analysis indicated that 2 missing studies had to be added, the analysis as a whole was not affected (RR 1.0451, 95% CI [0.5639, 1.9371], I^2^ 0%, *P* = .8885). The cumulative meta-analysis demonstrated that omitting the study conducted by Wang et al^[10]^ led to an obvious increase in the RR from 1.21 to 1.81, but did not change the final results. Thus, although this most recent RCT might not change the morbidity, it could make a stronger contribution to support our conclusions.

### 3.7 Need for permanent pacemaker

**Figure 6:**
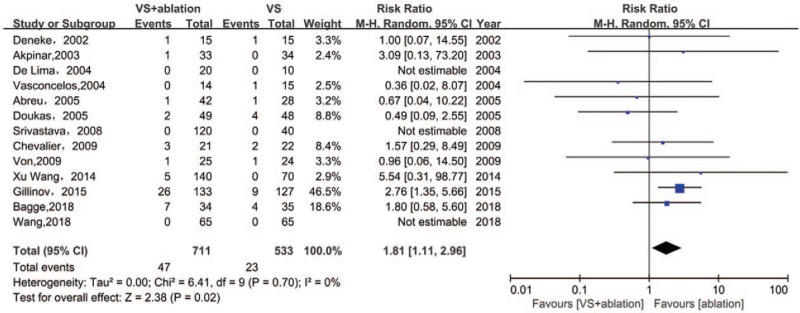
Forest plot of permanent pacemaker requirement, showing summary of RR with 95% CI for included studies. CI = confidence interval, RR = relative risk, VS = valvular surgery.

The use of temporary pacemakers largely depended on the decision of surgeons; thus, this factor was not considered in our analysis. The use of a permanent pacemaker (PPM) indirectly reflected an improvement in SR after valvular surgery. Some surgeons believed that the ablation procedure could prevent multiple recurrences, which might impact cardiac conduction to some extent. We found significant differences between the surgical valvular treatment associated with the ablation group and the group with valvular surgery alone (RR 1.84, 95% CI [1.15, 2.95], I^2^ 0%, *P* = .01). Interestingly, we identified 3 studies using the trim and fill analysis, which could improve significance (RR 2.2192, 95% CI [1.3629, 3.6135], I^2^ 4.2%, *P* = .001) (Fig. 6).

### 3.8 Bleeding

**Figure 7:**
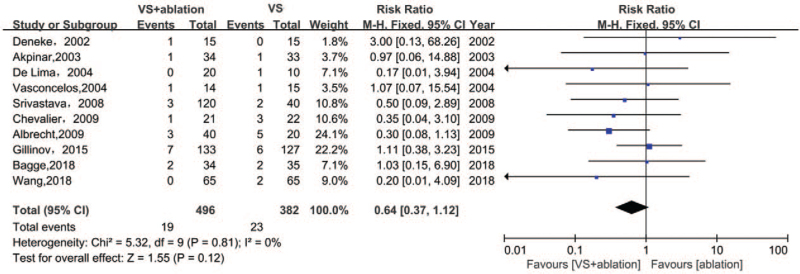
Forest plot of the incidence of bleeding after surgery, showing summary of RR with 95% CI for included studies. CI = confidence interval, RR = relative risk, VS = valvular surgery.

Bleeding-related events in the 2 groups were analyzed among 878 patients in 10 studies (Fig. 7). No significant differences were found with respect to bleeding between both groups (3.8% vs 6.0%, RR 0.64, 95% CI [0.37, 1.12], I^2^ 0%, *P* = .12). The funnel plot showed symmetry and the score from Egger analysis was *P* = .6915, which confirmed a low risk of bias and heterogeneity. The cumulative meta-analysis indicated that earlier studies reported a higher risk of bleeding in the VSA group, probably due to poor mastery of the ablation procedure by surgeons.

### 3.9 Heart failure

Heart failure was found to be a common complication after cardiac surgery. Only 4 studies provided evidence with respect to this outcome. A total of 24 (of 281) and 21 (of 275) patients suffered from heart failure in the VSA and VS groups, respectively. The meta-analysis showed no significant differences between both groups (RR 1.11, 95% CI [0.63, 1.93], I^2^ 0%, *P* = .72). The trim and fill method suggested that 2 studies should be added, even after which, the effect size was unchanged (RR 1.37, 95% CI [0.82, 2.30], I^2^ 0%, *P* = .23).

### 3.10 Low cardiac output syndrome

Four studies including 456 patients reported the incidence of low cardiac output syndrome. There were no significant differences between the 2 groups, which was a trend similar to that observed in heart failure (RR 1.41, 95% CI [0.57, 3.46], I^2^ 18%, *P* = .46). The morbidity due to low cardiac output syndrome was 10 in 268 (VSA group) and 6 in 188 (VS group). One study had to be added to decide the total effect size (RR 0.83, 95% CI [0.21, 3.32], I^2^ 38%, *P* = .79) based on the trim and fill analysis. No changes were observed compared with the original results.

## 4 Discussion

Patients with heart valve disease exhibit a higher prevalence of AF compared with other heart diseases. In recent decades, valvular surgery concomitant with AF ablation has been a common procedure for these patients. The treatment goal in AF includes the reduction of the incidence of stroke, reducing symptoms by controlling the heart rate, and decreasing the occurrence of tachycardia combined with myocardiopathy. Antiarrhythmic drugs are associated with some life-threatening side effects; thus, non-pharmacological methods have been gaining increased attention. Based on the continuous understanding of the mechanisms of AF, surgical treatments have been developed to restore SR, among which Maze is a well-known procedure. The main purpose of Maze is to block reentry to restore SR, maintain conduction of the sinoatrial and atrioventricular nodes to maintain the atrioventricular sequence, and maintain the mechanical structure of atria to improve hemodynamic function.

This meta-analysis based on RCTs revealed that valve surgery in combination with ablation could be effective in increasing the chances of restoring SR, irrespective of the time (discharge, 3–6 months, ≥12 months) after surgery chosen in this study to compare with valve surgery only. Even though publication bias was significantly different in SR restoration at discharge, it did not influence the results. As discussed earlier, this bias might be due to the variation in surgical methodology. Mitral valve surgery was safe and effective in patients in restoring SR by using ablation to treat AF.^[24–26]^ There is no doubt that surgical ablation can successfully block AF conduction to maintain the normal SR, as reported in previous reviews. Phan et al^[27]^ and McClure et al^[6]^ evaluated the efficacy of promoting SR in patients who underwent cardiac surgery combined with surgical ablation. In this meta-analysis, we focused on patients who underwent valve surgery owing to the higher susceptibility of VHD to AF. Therefore, surgical ablation could be effective in valvular surgery patients.

The findings of this systematic review demonstrated that ablation combined with valvular surgery did not increase the risk of postoperative stroke or death. Even if extra time was spent performing the ablation technique, CPB and AOC time was prolonged in the VSA group and did not appear to add to the morbidity of stroke and death, as determined in this meta-analysis. CPB-induced ischemia-reperfusion injury was the consequence of a series of systemic inflammatory reactions. Due to the extension and amplification of the inflammation cascade, multiple organs and systems were affected, resulting in coagulation, respiratory, and cardiac dysfunction. However, it was interesting to note the observed outcome that prolonged CPB time did not actually increase all-cause mortality. On one hand, it was probably because increasing the CPB time was not enough to cause an adverse effect. On the other hand, it was attributed to the therapeutic efficacy of surgical ablation, which decreased AF-related mortality. The benefits from surgical ablation counteract the adverse effects resulting from prolonged CPB time; thus, all-cause mortality was not significantly different between the 2 groups.

Our findings differed from those in the systematic reviews by Phan et al^[27]^ and McClure et al,^[6]^ which confirmed no significant increases in the PPM requirement in the overall population that underwent the ablation procedure. Our results contradicted their published findings. The utilization of a PPM in patients in the VSA group was 1.84 times higher compared with that in the VS group. After adjusting using the trim and fill methods, the relative risk was found to increase to 2.2. Irrespective of publication bias, the need for a PPM was higher in the VSA group. A retrospective study conducted on 87,426 patients who underwent surgical valve replacement revealed that AF ablation would not increase the risk of mortality and stroke, but indicated the requirements of a pacemaker.^[28]^ This retrospective study with a large sample size explored an idea similar to ours. We not only used the fixed-effect model but also the relatively conservative random-effect model to evaluate the overall effect size, which revealed a similar significance (random-effect model: RR 1.81, 95% CI [1.11, 2.96], I^2^ 0%, *P* = .02). Therefore, the results from this meta-analysis were appropriate. Compared with the study by Phan et al,^[27]^ we could conclude based on our cumulative meta-analysis that the effect size had been retroflected after the inclusion of the study by Gillinov et al,^[12]^ which had a higher weightage among the included studies. Additionally, the results were based on the odds ratio, which might be conservative in underestimating the overall effect size. In this review, RR was appropriately used to assess the effect size and we arrived at a positive conclusion. McClure et al^[6]^ suggested a higher PPM requirement in biatrial ablation not but for left-sided ablation only using subgroup analysis, but the total PPM requirement was not significantly different. Their research objects focused on adults with AF undergoing cardiac surgery and, therefore, had a wider target range. For example, patients who only underwent CABG were included in their review; otherwise, patients with coronary heart disease might be less susceptible to bradyarrhythmia than VHD. We have provided the latest evidence-based findings regarding the need for a PPM in valvular surgery patients.

Among VHD patients accepted for valvular surgery with or without the ablation procedure, the hospital length of stay showed a similar trend. The additional procedure increased CPB time, but no further complication or increase in hospitalization was noted. The morbidity of bleeding, heart failure, and low cardiac output syndrome did not show an increase in the VSA group. Therefore, surgical ablation could be safe for patients undergoing valvular surgery.

To the best of our knowledge, this is the first meta-analysis to report the efficiency and safety of surgical ablation for the treatment of AF in patients undergoing valvular surgery. This is also the first meta-analysis that is completely based on RCTs related to this field. Previous related meta-analyses might have been forced to consider many retrospective studies. Thus, as a consequence of the lack of RCTs, we were made available some low-quality evidence. We found high heterogeneity in the restoration of SR at discharge. Surgical technique, energy choice, surgical lesions, or methods of SR assessment were the factors responsible for the induction of high heterogeneity. Given the current state in the operating room, we assume that the comparison is valid at this time.

## 5 Conclusions

This meta-analysis is most likely the most comprehensive study on the safety and efficacy of AF ablation in patients undergoing valvular surgery. Based on rigorous analysis, we could conclude that AF ablation was an effective and safe procedure for patients undergoing valvular surgery; otherwise, to some extent, the use of a PPM was found to be higher. The controversy of rhythm disorders associated with the use of PPMs has been ongoing for decades. This meta-analysis once again refuted some recent views and confirmed that ablation promoted the demand for PPM in valvular surgery patients. A few side effects of ablation on cardiac conduction in these individuals were identified. Additional multicenter RCTs enrolling larger patient populations should be conducted to support this opinion. We are likely to pursue this aspect in our future studies.

### 5.1 Limitation

RR and *P* value determinations were made using Review Manager and R. The results might be a consequence of the different definitions of MD. R depends on the standard MD, whereas Review Manager relies on the weighted MD. However, these results did not significantly impact our results. Therefore, we believe that the conclusions derived from our meta-analysis are reliable.

## Author contributions

**Data curation:** Yu Zhang.

**Formal analysis:** Xiaochu Wu.

**Investigation:** Tianyao Zhang, Xiaochu Wu, Yu Zhang.

**Methodology:** Tianyao Zhang, Xiaochu Wu, Lin Zeng, Bin Liu.

**Supervision:** Bin Liu.

**Writing – original draft:** Tianyao Zhang.

**Writing – review & editing:** Lin Zeng, Bin Liu.
